# Immunostimulatory Pickering emulsion for oral vaccine delivery

**DOI:** 10.1016/j.ijpharm.2025.125890

**Published:** 2025-06-23

**Authors:** Jin Xie, Xiaodi Li, Grahmm A. Funk, Su Jeong Song, Udita Shah, Connor S. Ahlquist, Hyunjoon Kim

**Affiliations:** a Department of Pharmaceutical Chemistry, University of Kansas, Lawrence, KS 66047, USA; b Bioengineering Program, University of Kansas, Lawrence, KS 66047, USA

**Keywords:** Oral vaccine, Pickering Emulsion, Nanoparticle

## Abstract

To overcome gastric acid degradation and ensure robust immune activation, a novel Pickering emulsion stabilized by poly(lactic-co-glycolic acid) (PLGA) nanoparticles was developed for the co-delivery of vaccine antigens and adjuvants via the oral route. Pickering emulsions, stabilized by solid particles, can enhance stability and protect antigens from gastric degradation. We encapsulated a TLR7/8 agonist R848 in PLGA nanoparticles and fabricated Pickering emulsions (R848-PLGA-NP@PE) to boost immune activation, and further prepared model antigen Ovalbumin (OVA) loaded Pickering emulsion formulation (R848-PLGA-NP@PE-OVA) to induce antigen-specific immune responses. R848-PLGA-NPs can improve vaccine efficacy by serving both as a stabilizer and an adjuvant, activating antigen-presenting cells (APCs). R848-PLGA-NP@PE-OVA exhibited a uniform particle size (245 nm), stable zeta potential (− 40 mV), and high antigen encapsulation efficiency (*>*80 %), that were tested in Simulated Intestinal Fluid (SIF) and Simulated Gastric Fluid (SGF). R848-PLGA-NP@PE exhibited enhanced uptake by and activation of dendritic cells compared to control groups. In vivo, R848-PLGA-NP@PE significantly improved CD4 + T cell, CD8 + T cell, and NK cell activation. Notably, granzyme B expression in NK cells reached 2.1 times the level of the PBS group and 1.45 times that of the Free OVA + R848 group. The OVA-specific IgG level in the R848-PLGA-NP@PE-OVA group was approximately 3.9 times that of the PBS group and 2.5 times that of the free R848 + OVA group. Fecal OVA-specific IgA levels were significantly higher than control group. The combined data suggests that Pickering emulsions fabricated with PLGA-NPs are versatile oral vaccine delivery platforms to induce cellular and humoral immune responses.

## 1. Introduction

Vaccines have significant contribution to global public health by preventing and controlling infectious diseases such as influenza and polio. Injectable vaccines, delivered via intramuscular (IM), subcutaneous (SC), and intradermal (ID) routes, have been widely adopted due to their ability to elicit robust immune responses. However, injection-based delivery methods present certain limitations, including the need for a trained healthcare personnel (Harb et al., 2015), cold chain storage (Lin et al., 2020), and injection-associated discomfort, which may limit patient compliance and accessibility (Taddio et al., 2012). However, oral vaccines, offering needle-free administration, present a promising alternative by improving patient compliance and facilitating a quick and convenient distribution, especially in resource-limited areas (Russell-Jones, 2000). Oral vaccines are particularly effective for gastrointestinal (GI)-tract resident pathogens, such as polio (Blume and Geesink, 2000), cholera (Howard, 1971), rotavirus (Clark et al., 2019), and typhoid (Syed et al., 2020). Moreover, oral vaccines can induce mucosal immunity characterized by IgA that is essential to fight pathogens entering through the GI and respiratory tracts.

Despite these advantages, oral vaccines face significant challenges that limit their clinical application. A major hurdle is GI degradation, as gastric acid and digestive enzymes can break down antigens before vaccine components reach the intestinal immune cells, reducing vaccine potency (Vela Ramirez et al., 2017). Efficient antigen absorption and immune activation are further hindered by the intestinal epithelial barrier, which restricts antigen passage and uptake by immune cells (Chen et al., 2019). Consequently, insufficient antigen uptake and degradation results in sub-optimal immune responses. As a result, most clinically approved oral vaccines require multiple doses for effective immunity. For example, the oral poliovirus vaccine (OPV) typically requires three primary doses, wit additional doses recommended in high-risk areas (Moriniere et al., 1993). The oral Ty21, a typhoid vaccine follows a three-dose regimen on alternate days, with booster doses needed every five years for high-risk individuals (Simanjuntak et al., 1991). Similarly, the oral rotavirus vaccine is administered in a three-dose schedule at 6, 10, and 14 weeks of age (Velasquez-Portocarrero et al., 2022). These challenges suggest an urgent need for an optimized oral vaccine delivery strategy that can protect the antigen from degradation and elicit a robust immune response.

Various drug delivery systems have been explored to enhance antigen protection and facilitate transport across the mucosal barrier. Strategies such as biodegradable nanoemulsions (Kocabiyik et al., 2024), engineered nano-cellulose bio-composites (Cao et al., 2023), biomimetic micromotors (Wei et al., 2019), and Pickering emulsions (Li et al., 2023) were employed to shield antigens from enzymatic degradation, improve GI retention, and enhance antigen uptake by immune cells. Recently, Pickering emulsions have gained attention as a promising platform for vaccine delivery due to their structural and physicochemical properties. Stabilized by solid particles, Pickering emulsions exhibit high flexibility and deformability (Staats and Burkhart, 2018). This characteristic not only facilitates retention at the administration site but also promotes efficient antigen uptake and activation of APCs (Xia et al., 2018). Furthermore, the particle-stabilized interface provides a large specific surface area, enhancing interactions between APCs and target molecules or receptors, thereby improving immune recognition and response (Du et al., 2022). Hence, we propose using Pickering emulsions stabilized with biocompatible polymeric nanoparticles as an oral vaccine delivery platform as they can provide a barrier against gastric acid and allow controlled antigen release (Li et al., 2023; Yu et al., 2024). Particularly, we fabricated nanoparticles with PLGA, an FDA-approved biodegradable and biocompatible polymer (Danhier et al., 2012) to formulate Pickering emulsions.

APC activation is also critical for successful oral vaccination as APC activation enhances cross-presentation of antigens to T cells that leads to T cell priming and activation. It also facilitates antigen presentation to follicular helper T cells (Tfh cells) that drive B cell activation, leading to antibody production, affinity maturation, and class-switch recombination, thereby APC activation induces both cellular and humoral immune responses. Resiquimod (R848), a TLR7/8 agonist can enhance maturation and antigen-presenting capability of DCs and macrophages and therefore can be an efficient oral vaccine adjuvant (Chao et al., 2024; Wagner et al., 2021; Zhang et al., 2021). However, the poor solubility of R848 in the aqueous solution poses a challenge for its effectiveness towards vivo application. To address this, we encapsulated R848 within PLGA nanoparticles (R848-PLGA-NP) which can be dispersed in normal saline without organic solvents. In addition to enhancing solubility of R848, PLGA nanoparticles serve as effective stabilizers for Pickering emulsions, providing structural stability and enabling the controlled release of the encapsulated antigen-adjuvant system (Jiao et al., 2022; Zhang et al., 2022).

In this study, we designed and fabricated a Pickering emulsion-based oral vaccine platform using squalene as the oil phase and R848-loaded nanoparticles (R848-NPs) as both stabilizer and adjuvant. This platform was designed to efficiently co-deliver the antigen and adjuvant, to enhance APC activation, and subsequently elicit cellular immunity (e.g., T cell and NK cells), as well as humoral immunity (e.g., serum OVA-specific IgG, fecal OVA-specific IgA) offering a promising strategy for effective oral vaccination.

## 2. Materials and methods

### 2.1. Materials

Poly(lactic-co-glycolic acid) ester end-capped (PLGA, 50:50 LA:GA, Mw: 45,000–55,000 Da) and Poly(lactide-co-glycolide)-b-poly(ethylene–glycol)-carboxylic acid (PLGA-PEG-COOH, Mw: 10,000–2,000 Da, LA: GA 50:50) were purchased from Polyscitech (West Lafayette, IN, US), Resiquimod was ordered from MedChemExpress (Monmouth Junction, NJ, USA), Albumin from chicken egg white (ovalbumin, OVA), Dimethyl sulfoxide (DMSO), squalene were purchased from Sigma-Aldrich (St. Louis, MO, USA), Ovalbumin, Alexa Fluor^™^ 647 Conjugate (OVA-647) was purchased from Invitrogen (Waltham, MA, USA). Flow cytometry antibodies were purchased from Biolegend (San Diego, CA).

### 2.2. Animals

Female immunocompetent C57BL/6 mice (7–8 weeks old) were obtained from Charles River Laboratories (Wilmington, MA, USA) and housed in facilities managed by the Research Animal Resources Center at the University of Kansas. All experimental procedures involving animals were conducted in compliance with protocols approved by the Institutional Animal Care and Use Committee (IACUC) of the University of Kansas (AUS 281–01).

### 2.3. Preparation of nanoparticles and emulsions

R848-loaded PLGA nanoparticles (R848-PLGA-NPs) were synthesized using the nanoprecipitation method (Du et al., 2022). Briefly, 15 mg of PLGA, 5 mg of PLGA-PEG-COOH, and 4 mg of R848 were dissolved in 1 ml DMSO and subsequently added dropwise to 10 ml DI water under constant stirring. The resulting mixture was allowed to stabilize for 1 h. The nanoparticles were purified by centrifugation at 4000 rpm for 15 min using an Amicon Ultra centrifugal filter (30 kDa) to remove residual DMSO and other impurities. Finally, the particles were resuspended in deionized (DI) water for subsequent applications. R848-PLGA nanoparticles were dispersed in methanol and sonicated for 30 min to extract the encapsulated drug. The resulting solution was filtered through a 0.45 μm membrane filter and analyzed for R848 content using HPLC, following previously reported methods (Kim et al., 2018).

The Pickering emulsion was prepared using a sonication method (Du et al., 2022). In brief, 1.5 ml solution of R848-PLGA-NPs (0.4 %) were dispersed in the aqueous phase and subsequently mixed with 125 μl of squalene, which was used as the oil phase. The mixture was then sonicated at 90 % amplitude for 1 min using a 6-second on, 1-second off pulse cycle (QSONICA Sonicator). After sonication, 3 mg of Ovalbumin (OVA) was added, followed by a second round of sonication under the same conditions for an additional minute to form R848-NP@PE-OVA. For fluorescent tracking purposes, OVA-647 was used as a substitute for OVA to prepare the fluorescently labeled R848-NP@PE-OVA. The morphology of R848-PLGA-NP and R848-PLGA-NP@PE were analyzed using transmission electron microscopy (TEM) (Hitachi H-8100, Hitachi, Japan). The samples were diluted in distilled water and stained with 2 % phosphotungstic acid (PTA) at a 1:1 vol ratio. A droplet of the stained sample was placed on a copper grid and allowed to dry at room temperature before examination under the electron microscope.

### 2.4. Characterization of R848-NP@PE-OVA

The size and zeta potential of R848-NP@PE-OVA were measured using a dynamic light scattering (DLS) device (Zetasizer Nano-ZS, Malvern). The amount of R848 loaded into the nanoparticles was quantified by HPLC as previously reported (Kim et al., 2018). The structural properties of the dried Pickering emulsion were analyzed using Fourier transform infrared spectroscopy (Nicolet^™^ Summit^™^ FTIR Spectrometer) over a range of 400–4000 cm. The stability of R848-NP@PE-OVA was assessed by storing the samples at 4 ^◦^C, with aliquots taken on days 1, 3, 5, and 7 for analysis of particle size, zeta potential, and drug encapsulation efficiency. The stability of R848-NP@PE-OVA in biological fluids was assessed by dispersing the samples in Simulated Gastric Fluid (SGF) and Simulated Intestinal Fluid (SIF) and incubating them in a shaking incubator (100 rpm, 37 ^◦^C). SGF was prepared with 2 g/L NaCl (pH 1.4), with or without 3.2 g/L pepsin. SIF was prepared with 6.8 g/L KH_2_PO_4_ (pH 6.8), with or without 10 g/L pancreatin (Kim et al., 2020). At designated timepoints, the particle size and zeta potential were measured. OVA encapsulation efficiency was determined using an Amicon^®^ Ultra centrifugal filter (50 kDa MWCO). The formulation was centrifuged at 4000 rpm for 15 min to separate free OVA. The amount of unencapsulated OVA in the filtrate was then quantified using a BCA protein assay kit (Thermo Scientific). In addition, SDS-PAGE was conducted to assess the structural stability of R848-PLGA-NP@PE-OVA and free OVA in SGF (with or without pepsin) and SIF (with or without pancreatin). Each sample was mixed with Tris-Glycine SDS sample buffer and NuPAGE^™^ sample reducing agent, then heated at 80 ^◦^C for 10 min. Subsequently, 10 μL of each sample was loaded onto 4 %–20 % polyacrylamide gels and run in a Mini Gel Tank (Invitrogen) at a constant voltage of 225 V for 35 min.

The in vitro release profiles of OVA and R848 from R848-PLGA-NP@PE-OVA were evaluated using a dialysis-based method adapted from previous reports (Kim et al., 2018). For OVA release, OVA-647 was encapsulated, and the release was monitored by measuring its fluorescence intensity. R848-PLGA-NP@PE-OVA formulations were dispersed in SGF or SIF and transferred into dialysis tubes (Float-A-Lyzer G2, 50 kDa molecular weight cutoff). The dialysis tubes were immersed in 7 mL of release buffer placed inside 50 mL plastic tubes and maintained in a water bath shaker at 37 ^◦^C and 100 rpm. At predetermined time points, the entire release medium was withdrawn and replaced with fresh buffer. The amount of R848 released was quantified by HPLC, while the release of OVA-647 was determined by measuring fluorescence intensity (excitation at 650 nm, emission at 668 nm) using a BioTek Cytation 7 plate reader.

### 2.5. Uptake of R848-NP@PE-OVA by bone marrow-derived dendritic cells (BMDCs)

BMDCs were generated from bone marrow cells isolated from the femurs and tibiae of female C57BL/6 mice, following established protocols (Kim et al., 2018). To study the cellular uptake of R848-PLGA-NP@PE-OVA by BMDCs, BMDCs (1 × 10^6 cells/well) were seeded in a 48-well cell culture plate (200 μl/well). The next day, Free OVA-647 and R848-PLGA-NP@PE-OVA647 (5 μg/ml OVA-647) were added to the BMDCs and incubated for 2 h. Cells were then detached using a non-enzymatic cell dissociation solution, then washed twice with phosphate-buffered saline (PBS). Subsequently, the cells were stained with an anti-mouse CD11c Antibody. The cellular uptake of OVA by BMDCs was determined by measuring the Alexa Fluor^™^ 647 fluorescence signal from CD11c-positive cells using flow cytometry method (Cytek Aurora).

To further examine the internalization of R848-PLGA-NP@PE-OVA by BMDCs, BMDCs were incubated with free OVA-647 or R848-PLGA-NP@PE-OVA647 for 2 h. After incubation, the cells were gently washed twice with PBS. The cells were then stained with Hoechst 33,342 (blue, Thermo Fisher) for 15 min to visualize the nucleus. Subsequently, the cells were washed twice with PBS, followed by the addition of 200 μl PBS, and images were captured using the BioTek Cytation 7 imaging system.

### 2.6. In vitro BMDCs activation and cell viability

BMDCs (1 × 10^6 cells/well in 200 μl) were seeded into a 48-well cell culture plate. After cell attachment, complete medium containing PBS, free OVA, free OVA + R848, or R848-PLGA-NP@PE-OVA, each at an equivalent concentration of OVA (1 μM) and R848 (1.7 μg/ml), was added to the respective wells. After 24 h of incubation, BMDCs were detached and stained with anti-mouse CD11c-APC-Cyanine 7, anti-mouse CD80-FITC, and anti-mouse CD86-Alexa Fluor-700. The expression of co-stimulatory molecules was analyzed using flow cytometry (Cytek Aurora).

The cytotoxicity of R848-NP@PE-OVA was assessed in BMDCs using the LDH Cytotoxicity Assay (Thermo Fisher). BMDCs were seeded at a density of 1 × 10^5 cells per well in 100 μl of culture medium and incubated for 2 h. Subsequently, 100 μl of culture medium containing varying concentrations of R848-NP@PE-OVA was added to each well. After 24 h of incubation, the supernatants were collected, and cytotoxicity was evaluated in according with the manufacturer’s protocol.

### 2.7. In vivo immunization

C57BL/6 mice were divided into three groups: PBS group, Free OVA + R848 group and R848-PLGA-NP@PE-OVA group. The mice were immunized on Days 1 and 7 with 100 μl of free OVA + R848 solution (100 μg OVA + 20 μg R848), R848-PLGA-NP@PE-OVA solution (100 μg OVA + 20 μg R848), or an equivalent volume of PBS. On Day 11, the mice were sacrificed, and blood, fecal samples, and spleens were collected.

#### 2.7.1. T Cell, NK Cell, and dendritic cell response in the spleen

Spleens were collected and placed in PBS, then mechanically homogenized using the gentleMACS^™^ Octo Dissociator with Heaters (Miltenyi Biotec). The resulting cell suspensions were filtered through 70-μm strainers to remove debris. The samples were centrifuged, and the cell pellet was resuspended in PBS containing 1 mM ethylenediaminetetraacetic acid (EDTA) and 1 % fetal bovine serum (FBS). The cells were incubated at room temperature for 15 min followed by an additional centrifugation cycle. Following centrifugation, the supernatant was removed, and lysis buffer was added to the cell pellet to lyse red blood cells. After a 5-minute incubation at room temperature, the samples were centrifuged to remove supernatant, and the cell pellet was resuspended in PBS. To prevent nonspecific binding, the cells were first blocked with anti-mouse CD16/32 antibody. To characterize T and NK cells, the cells were stained with the following fluorophore-conjugated antibodies: CD45-PerCP, CD3-FITC, CD49b-Alexa Fluor^®^ 647, CD4-violetFluor^™^ 450, CD8a-Alexa Fluor^®^ 700, CD44-PE-Cyanine7, CD69-PE, and Granzyme B Recombinant-PE/Dazzle^™^ 594. For dendritic cell (DC) identification, the cells were stained with CD45-PerCP, CD11c-APC-Cyanine7, CD80-FITC, CD86-Alexa Fluor^®^ 700, and CD40-PE. Stained cells were analyzed using flow cytometry (Cytek Aurora), and data were processed using FlowJo software (version 10.8.1).

#### 2.7.2. Serum OVA-specific IgG analysis

Serum IgG levels were measured using an established method with modification (Kim et al., 2023). Blood samples were centrifuged at 5000 rpm for 5 min, and the serum was isolated and stored at − 80 ^◦^C. OVA was dissolved in coating buffer at 10 μg/mL, and 100 μL was added to each well of a 96-well plate and incubated overnight. The plate was washed with PBS-T (PBS containing 0.2 % Tween 20) and blocked with PBS-T containing 10 % bovine serum albumin (BSA) for 1 h. The serum samples were diluted 1:400 in PBS-T, added to the wells, and incubated for 2 h. After washing, goat anti-Mouse IgG (H + L) Poly-HRP Secondary Antibody (ThermoFisher) was added and incubated at room temperature for 1 h. Unbound secondary antibody was removed by washing, followed by the addition of the HRP substrate solution. After a 20-minute incubation, absorbance was measured at 450 nm using a BioTek Cytation 7 plate reader.

#### 2.7.3. Mucosal OVA-specific IgA analysis

Mucosal IgA levels were measured using a modified protocol (Carlsen et al., 2023). Fecal pellets were collected and stored at −80 ^◦^C. Before analysis, the samples were thawed, homogenized in a buffer (PBS containing 0.1 mg/mL soybean trypsin inhibitor, 1 % BSA, 25 mM EDTA, 50 % glycerol, and 1 mM PMSF), vortexed thoroughly, and incubated at 4 ^◦^C with shaking at 100 rpm for 2 h. The homogenate was centrifuged at 12,000 × g for 10 min at 4 ^◦^C, and the supernatant was collected for further analysis.

A 96-well plate was coated with 100 μl of OVA (1 mg/mL in coating buffer) overnight at 4 ^◦^C. The plate was washed with PBS-T and then blocked with 10 % BSA in PBS-T for 1 h at room temperature. After washing, 100 μL of diluted fecal supernatants (1:50 in PBS-T) were added to each well and incubated for 2 h at room temperature. The plate was washed again, and 100 μL of goat anti-mouse IgA-HRP (ThermoFisher) was added and incubated for 1 h. After washing, HRP substrate solution (Biolegend) was added and incubated for 20 min in the dark. The reaction was stopped by adding 100 μL of 2 N sulfuric acid, and the optical density was measured at 450 nm.

### 2.8. Statistical analysis

Statistical analysis was performed using GraphPad Prism 10.0.0, and multiple groups were compared using one-way analysis of variance (ANOVA) with a *post hoc* Tukey test.

## 3. Results

### 3.1. Fabrication and characterization of R848-PLGA-NP

**Fig. 1.**
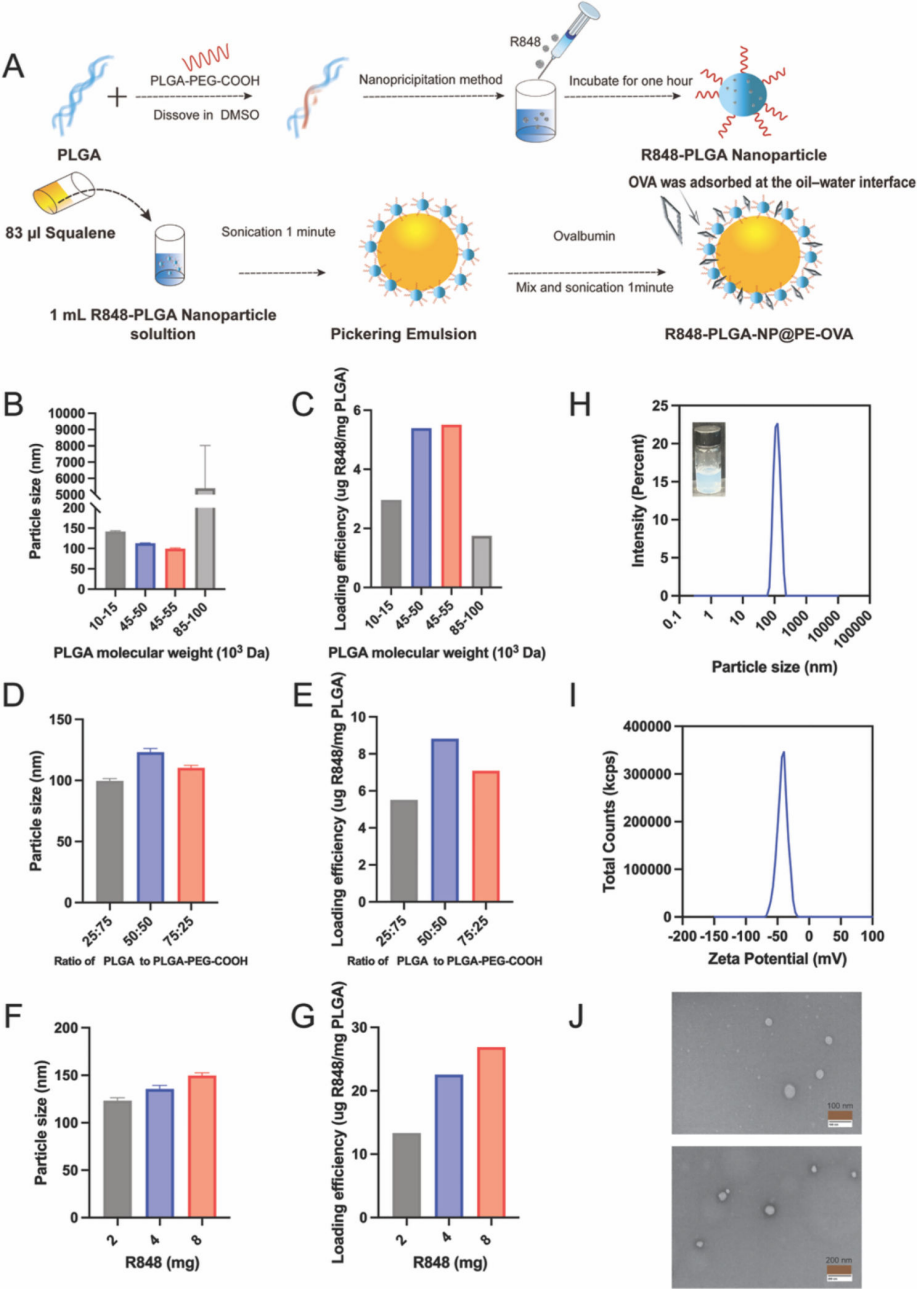
Optimization and Characterization of R848-PLGA-NP. (A) Schematic representation of the fabrication process of R848-PLGA-NP@PE-OVA. (B-G) Optimization of R848-PLGA-NP formulation. (B-C) Effects of different PLGA molecular weights on the particle size and loading efficiency of R848-PLGA-NP. (D-E) Influence of varying PLGA to PLGA-PEG-COOH ratios on the particle size and loading efficiency of R848-PLGA-NP. (F-G) Impact of different R848 concentrations on the particle size and loading efficiency of R848-PLGA-NP. (H-I) Particle size distribution and zeta potential of R848-PLGA-NP. (J) TEM morphology of R848-PLGA-NP. All data are represented as mean ± SD.

R848-PLGA-NP@PE-OVA formulation was optimized by fabricating a series of NPs fabricated with different PLGA molecular weight, PLGA to PLGA-PEG ratio, and concentration of R848 (Fig. 1 B–G), using the fabrication method described in Fig. 1A. The effect of PLGA molecular weight on the particle size and adjuvant loading efficiency of R848-PLGA nanoparticles (NPs) is presented in Fig. 1B and C. R848-PLGA-NP formulated with 85–100 kDa PLGA exhibited an exceptionally large size (~6000 nm), likely due to the increased viscosity of high-molecular weight PLGA, which impairs the diffusion of the organic phase into the aqueous phase, leading to the formation of large aggregates. Moreover, these nanoparticles exhibited a lower R848 loading efficiency compared to those prepared with 10–15 kDa, 40–55 kDa, and 45–55 kDa PLGA. In contrast, 45–55 kDa PLGA yielded the highest adjuvant loading efficiency, which was 1.8 times higher than that of 10–15 kDa PLGA and 3.14 times higher than that of 85–100 kDa PLGA, while also forming the smallest nanoparticles (99.57 ± 1.95 nm). Based on these findings, 45–55 kDa PLGA was selected for R848-PLGA-NP fabrication. To improve nanoparticle stability and dispersibility, PLGA-PEG-COOH was incorporated at varying ratios of PLGA to PLGA-PEG-COOH (75:25, 50:50, and 25:75). As shown in Fig. 1D and E, all three formulations exhibited comparable particle sizes. The 50:50 ratio resulted in the highest adjuvant loading efficiency; however, the difference compared to the 75:25 ratio was negligible. As higher PEG modification can seldom an trigger anti-PEG antibody response (Zhang et al., 2016), we selected 75:25 ratio. Additionally, the effect of the amount of incorporated R848 (2 mg, 4 mg, and 8 mg) on nanoparticle properties was evaluated (Fig. 1F and G). The 4 mg and 8 mg formulations produced R848-PLGA-NP with smaller sizes and higher adjuvant loading efficiencies compared to the 2 mg formulation. However, the difference in adjuvant loading between 4 mg and 8 mg was insignificant. To maximize efficiency while minimizing material use, 4 mg was selected as the optimal R848 input for the fabrication process. Based on the optimization study, 45–55 kDa PLGA, a PLGA to PLGA-PEG-COOH ratio of 75:25, and 4 mg of R848 were selected for the formulation of R848-PLGA-NPs, which were subsequently used for the preparation of Pickering emulsions. As shown in Fig. 1H and I, the optimized nanoparticles exhibited a uniform size distribution and a relatively narrow Zeta potential profile, indicating colloidal stability. TEM analysis (Fig. 1J) further confirmed that the R848-PLGA-NPs were nano-sized with an oval morphology and an average particle size of approximately 100 nm, consistent with the DLS measurements.

### 3.2. Fabrication and characterization of R848-PLGA-NP@PE-OVA

**Fig. 2.**
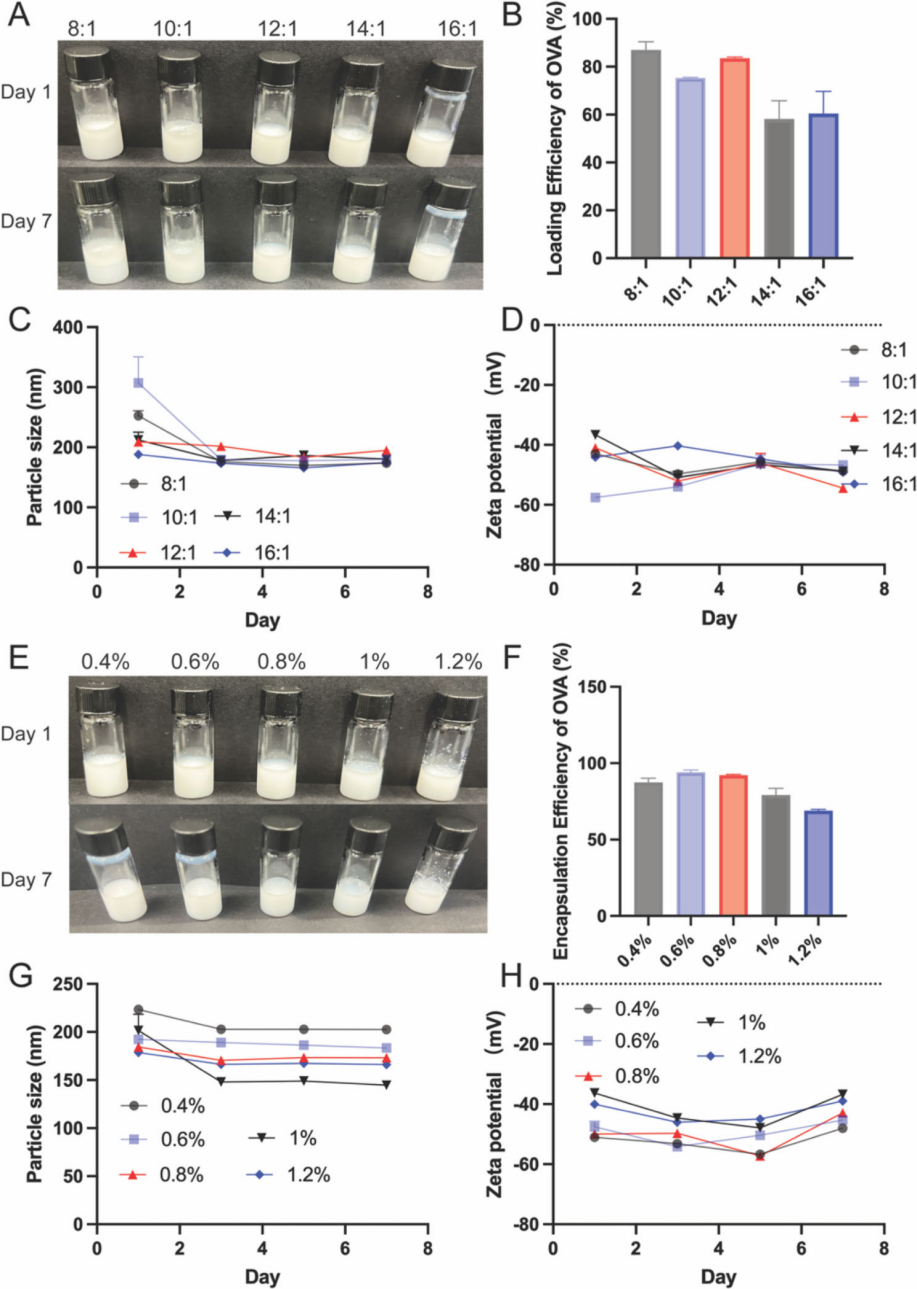
Optimization of oil-to-water ratio and R848-PLGA-NP concentration to fabricate R848-PLGA@PE-OVA. (A–D) Effects of varying oil-to-water ratios on the appearance, OVA encapsulation efficiency, particle size, and zeta potential of R848-PLGA-NP@OVA. (E–H) Effects of different particle concentrations on the appearance, OVA encapsulation efficiency, particle size, and zeta potential of R848-PLGA-NP@OVA. All data are represented as mean ± SD.

We subsequently investigated the effects of oil-to-water ratio and PLGA nanoparticle concentration on the visual stability, protein (OVA) loading efficiency, particle size, and zeta potential of the Pickering emulsion. Specifically, water-to-oil ratios of 8:1, 10:1, 12:1, 14:1, and 16:1 were tested on day 1, in which all emulsions appeared as uniform milky-white dispersions (Fig. 2A). However, after 7 days of storage at 4 ^◦^C, phase separation was observed in the 8:1 and 10:1 emulsion, likely due to the higher oil content exceeding the emulsifying capacity of the stabilizer, resulting in excess oil aggregation. In contrast, emulsions with 12:1, 14:1, and 16:1 ratios remained visually stable after storage. As shown in Fig. 2B, OVA encapsulation efficiency showed a trend consistent with the visual observations. The 12:1 group exhibited higher OVA loading than the 14:1 and 16:1 groups, suggesting that an overly low oil phase may reduce the available oil–water interface for OVA adsorption, which is critical for loading efficiency. Fig. 2C and D show that the particle size and zeta potential remained stable over 7 days of storage for all groups, with particle sizes of 200 nm and zeta potentials of –40 mV. Based on these results, we selected the 12:1 oil-to-water ratio for further experiments, which yielded a R848-PLGA-NP@PE-OVA with an OVA encapsulation efficiency of 83.5 % ± 0.49 %, a particle size of 209 ± 4.1 nm, and a zeta potential of –40.98 ± 0.40 mV. The TEM analysis of R848-PLGA-NP@PE-OVA (Supplementary Fig. 1) showed that the emulsion droplets were spherical in shape, with diameters ranging from 200 to 400 nm, consistent with the results of particle size measurements.

We then optimized the concentration of R848-PLGA-NP by preparing emulsions with particle concentrations of 0.4 %, 0.6 %, 0.8 %, 1.0 %, and 1.2 % (w/v). After 7 days of storage at 4 ^◦^C, the emulsions containing 0.4 %, 0.6 %, and 0.8 % PLGA-NPs remained stable and homogeneous, whereas phase separation and aggregation were observed in the 1.0 % and 1.2 % groups (Fig. 2E). This suggests that concentrations above 1.0 % may exceed the interface stabilization capacity, leading to NP sedimentation and instability. Consistently, OVA encapsulation efficiency (Fig. 2F) decreased when particle concentration exceeded 0.8 %, likely due to excessive PLGA-NPs interfering with OVA adsorption at the interface. Nonetheless, the particle size (Fig. 2G) remained stable at ~200 nm during storage, and the zeta potential (Fig. 2H) was maintained at approximately –40 mV across all concentrations. Thus, 0.4 % PLGA-NP was selected as the optimal concentration, yielding emulsions with a particle size of 223.3 ± 3.46 nm, a zeta potential of –51.01 ± 1.05 mV, and an OVA encapsulation efficiency of 87.45 % ± 2.70 %. Therefore, the final formulation of R848-PLGA@PE-OVA was prepared using an oil-to-water ratio of 12:1 and a PLGA-NP concentration of 0.4 %.

### 3.3. Stability and in vitro release of R848-PLGA-NP@PE-OVA

**Fig. 3.**
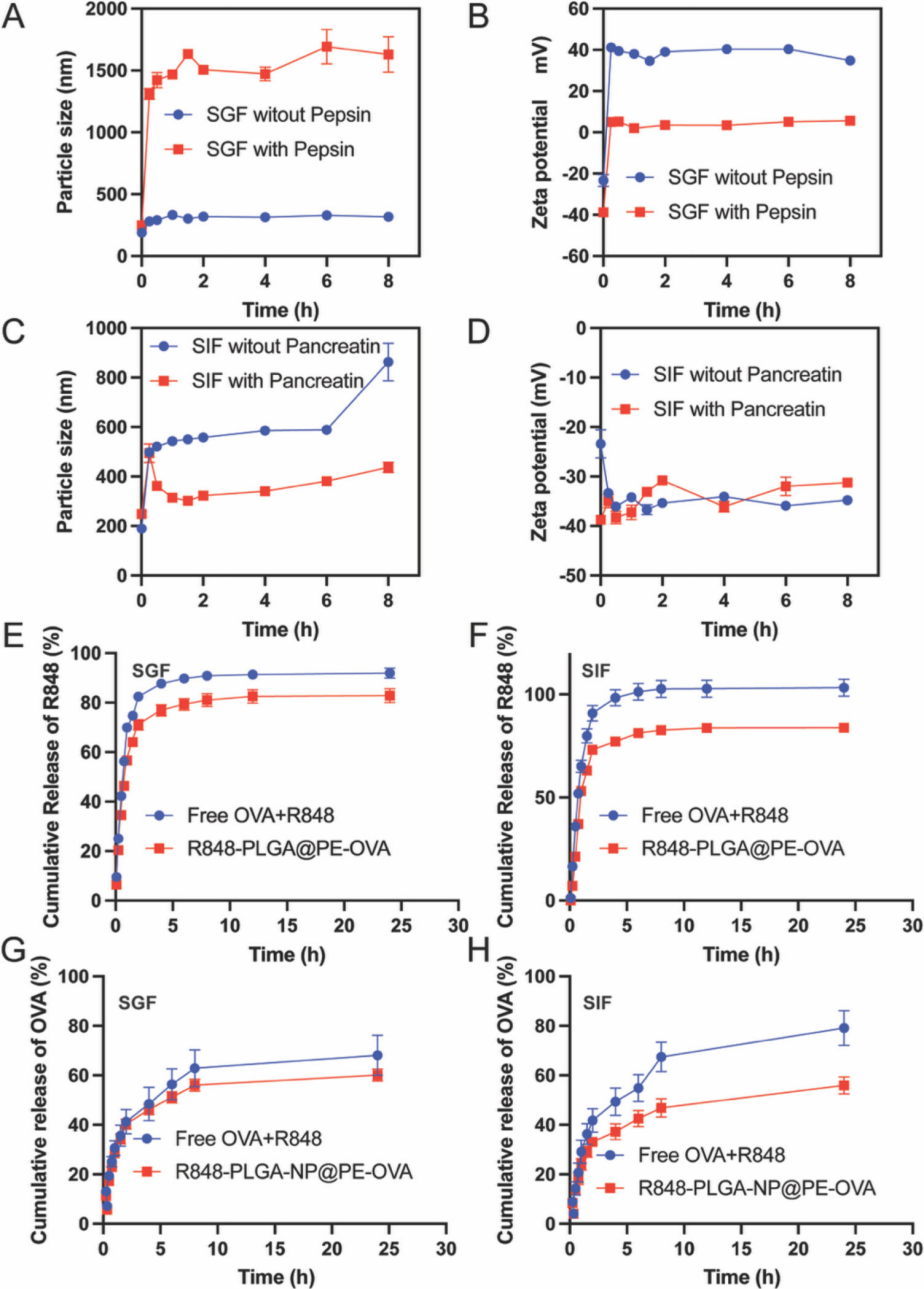
Stability and in vitro release profile of R848-PLGA-NP@PE-OVA (A-D) Stability assessment of R848-PLGA-NP@PE-OVA in SGF with or without pepsin and SIF with or without pancreatin by measuring particle size and zeta potential (E-F) In vitro release profiles of R848 in SGF and SIF. (G-H) In vitro release OVA in SGF and SIF. n = 3, All data are represented as mean ± SD.

To assess the stability of the Pickering emulsion in GI-tract, the optimized formulation was subsequently tested in SGF and SIF. In SGF without pepsin (Fig. 3 A), the particle size remained relatively stable over 8 h, increasing only slightly from 189 nm to approximately 300 nm. In SGF containing pepsin, the particle size increased more significantly compared to SGF without pepsin, which may be attributed to pepsin-induced aggregation of the R848-PLGA-NP@PE-OVA. The zeta potential shifted from − 26.8 mV to 40 mV within the first 15 min (Fig. 3B), which can be due to the adsorption of H^+^ ions onto the emulsion surface, leading to an overall positive charge. Following the initial shift, the zeta potential was stable at 40 mV throughout the 8 h. In SIF withour pancreatin (Fig. 3C and D), the particle size of the Pickering emulsion increased from 189.8 ± 2.4 nm to 497.7 ± 16.64 nm within the first 15 min, likely due to interactions between bile salts and the stabilizing nanoparticles at the oil–water interface, leading to surface restructuring and limited coalescence. However, after 6 h of incubation, the size stabilized at approximately 500 nm, suggesting that the R848-PLGA-NP@PE-OVA remains structurally stable under physiological conditions without significant aggregation or phase separation. Simultaneously, the zeta potential decreased from –23.38 ± 2.84 mV to –33.36 ± 0.51 mV within the first 15 min and remained stable at approximately − 35 mV over the subsequent 8 h. The increase in the absolute zeta potential of 10 mV suggests enhanced electrostatic stability of the Pickering emulsion in the SIF environment, which may contribute to improved dispersion and resistance to coalescence under intestinal conditions. In SIF with pancreatin, the particle size of R848-PLGA-NP@PE-OVA gradually increased from approximately 200 nm to over 400 nm within 8 h, while the zeta potential remained stable at around –30 mV, suggesting that pancreatin had minimal impact on both particle size and surface charge. As shown in Supplementary Fig. 2, SDS-PAGE was used to assess the structural stability of OVA. In the free OVA group, the band shifted downward after incubation with SGF (with or without pepsin), and disappeared after exposure to SIF with pancreatin, indicating degradation. Similar results were observed for PE-OVA. However, after incubation with SGF without pepsin, PE-OVA retained a band at ~45 kDa, while the free OVA band shifted, suggesting partial protection by the emulsion. Notably, under SGF with pepsin, PE-OVA maintained the 45 kDa band, whereas free OVA showed clear degradation, indicating improved resistance to enzymatic digestion.

To confirm the presence and successful encapsulation of both OVA and R848 within the formulation, FT-IR spectroscopy was conducted. Supplementary Fig. 3 shows the infrared spectra of free OVA, free R848, and R848-PLGA-NP@PE-OVA. In the spectrum of free OVA, characteristic absorption bands were observed at 1600–1700 cm^−1^ (C=O stretching), 1000–1200 cm^−1^ (C–N stretching), and 3200–3500 cm^−1^ (N–H stretching). For free R848, the spectrum displayed absorption bands at 1450–1600 cm^−1^ (C=C stretching), 700–900 cm^−1^ (aromatic C–H bending), and 3100–3500 cm^−1^ (N–H stretching). Minor variations in peak intensity may be attributed to slight drug loss during the Pickering emulsion fabrication process. The FT-IR spectrum of R848-PLGA-NP@PE-OVA displayed absorption peaks similar to free OVA and R848, confirming successful encapsulation.

To evaluate the in vitro release behavior of the R848 and OVA, the cumulative release profiles of R848 and OVA from R848-PLGA-NP@PE-OVA were investigated in simulated SGF and SIF. The cumulative release profiles of R848 from R848-PLGA-NP@PE-OVA in SGF and SIF are shown in Fig. 3E and F. In SGF, approximately 34.5% of R848 was released from R848-PLGA-NP@PE-OVA at 30 min, whereas 42.2 % of free R848 was released under the same conditions. After 8 h, 81.0 % of R848 was released from R848-PLGA-NP@PE-OVA, compared to 90.9 % release from the free drug. These results indicate that R848 exhibited a slower release from the nanoparticles compared to the free drug in SGF, suggesting that the nanoparticles provided a certain degree of protection for R848 in the acidic environment. In SIF, a similar delayed release pattern was observed. At 30 min, 21.3 % of R848 was released from R848-PLGA-NP@PE-OVA, while 35.98 % of free R848 was released. After 8 h, the cumulative release of free R848 reached 100 %, whereas the release from R848-PLGA-NP@PE-OVA was 82.65 %. These results further confirm that the nanoparticle formulation slowed the release of R848 in SIF compared to the free drug.

The cumulative release profiles of OVA from R848-PLGA-NP@PE-OVA in SGF and SIF are shown in Fig. 3G and H. In SGF, 19.3 % of free OVA was released at 30 min, compared to 17.3 % release from R848-PLGA-NP@PE-OVA. After 24 h, the cumulative release of free OVA was 68.1 %, whereas the release from R848-PLGA-NP@PE-OVA was 60.1 %. In SIF, at 30 min, the release of free OVA was 14.3 %, while the release from R848-PLGA-NP@PE-OVA was 13.56 %. After 8 h, free OVA exhibited a cumulative release of 67.4 %, compared to 46.8 % from R848-PLGA-NP@PE-OVA. Overall, these results indicate that both R848 and OVA exhibited slower release from R848-PLGA-NP@PE-OVA compared to their respective free forms in SGF, suggesting that the Pickering emulsion provided protective effects against acidic degradation. Moreover, in SIF, the R848-PLGA-NP@PE-OVA formulation demonstrated a sustained release profile for both R848 and OVA, which could be beneficial for oral vaccine delivery targeting the small intestine.

### 3.4. Cellular uptake of R848-PLGA@PE-OVA

**Fig. 4.**
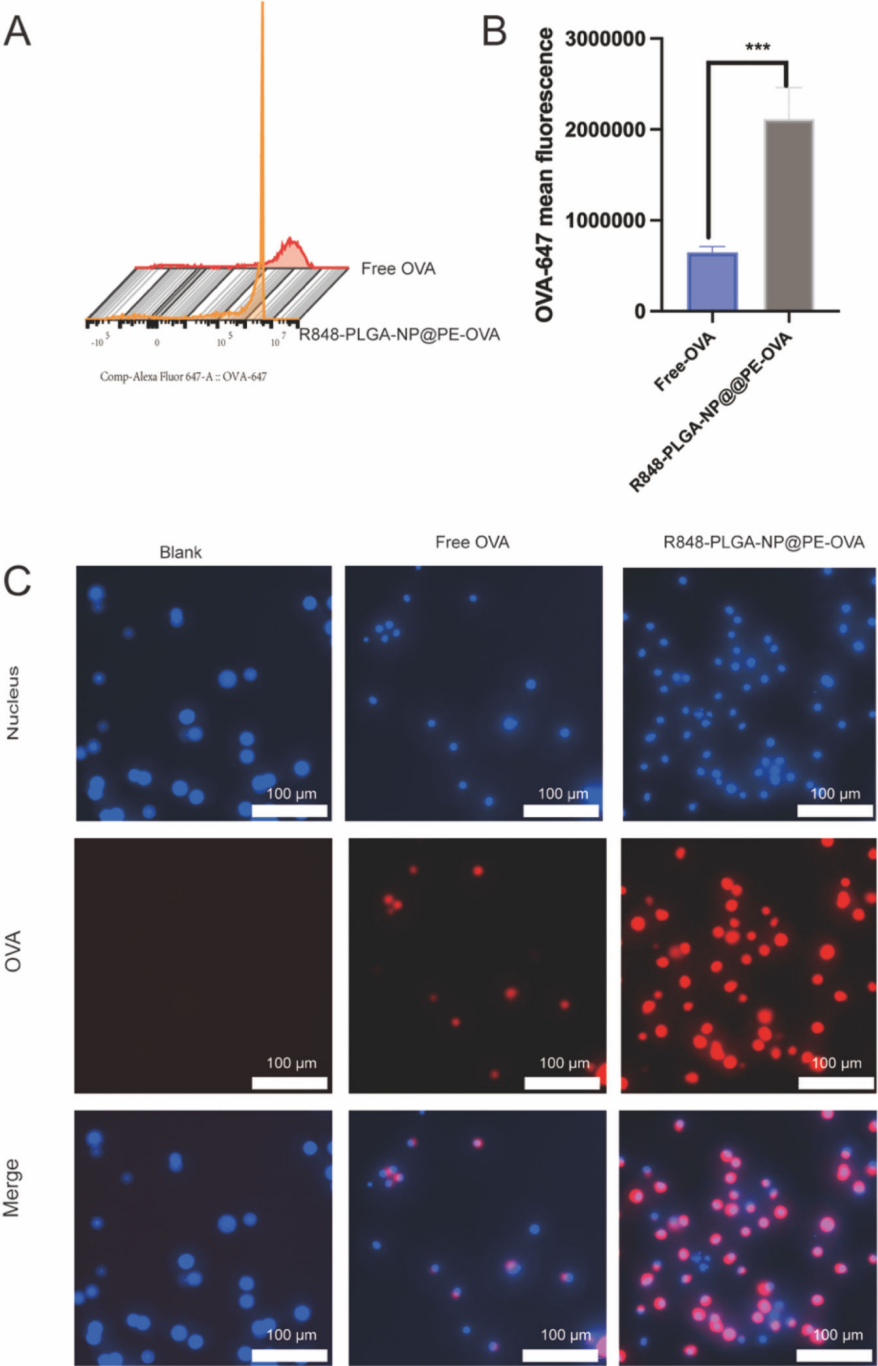
Uptake of R848-PLGA-NP@PE-OVA by BMDCs. (A) Histogram of OVA-647 signal intensities from BMDCs (B) Quantitative analysis of cellular uptake by flow cytometry (mean ± SD, n = 4), Student’s *t*-test ***P < 0.001 (C) Fluorescence microscopy images of BMDCs incubated for 2 h with free OVA, R848-PLGA-NP@PE-OVA, or PBS. Scale bar: 100 μm.

The efficient uptake of antigens by BMDCs is essential for antigen processing and presentation. To evaluate whether R848 encapsulated within PLGA-NP can enhance antigen uptake of BMDCs, we conducted BMDC uptake study using fluorophore labeled OVA (OVA-647). As shown in Fig. 4A and B, BMDCs treated with R848-PLGA-NP@PE-OVA showed OVA-647 intensity that was 3 times that of cells treated with free OVA-647, which is consistent with previous studies demonstrating the enhanced cellular uptake by DCs stimulated with TLR7/8 signaling (Toy et al., 2021). Fluorescence imaging further confirmed these findings, showing significantly higher OVA fluorescence intensity (red) in BMDCs incubated with R848-PLGA-NP@PE-OVA compared to those treated with free OVA (Fig. 4C). Collectively, these results indicate that R848-PLGA-NP@PE-OVA enhances OVA uptake by BMDCs.

Additionally, we evaluated the effect of R848-PLGA-NP@PE-OVA treatment on BMDCs viability (Supplementary Fig. 4). BMDCs treated with varying concentrations of R848-PLGA-NP@PE-OVA (corresponding to OVA concentrations ranging from 2 μM to 1 nM and R848 concentrations ranging from 28 μM to 200 nM) exhibited nearly 100 % viability. These results indicate that R848-PLGA-NP@PE-OVA is well-tolerated by BMDCs.

### 3.5. In vitro BMDC activation

**Fig. 5.**
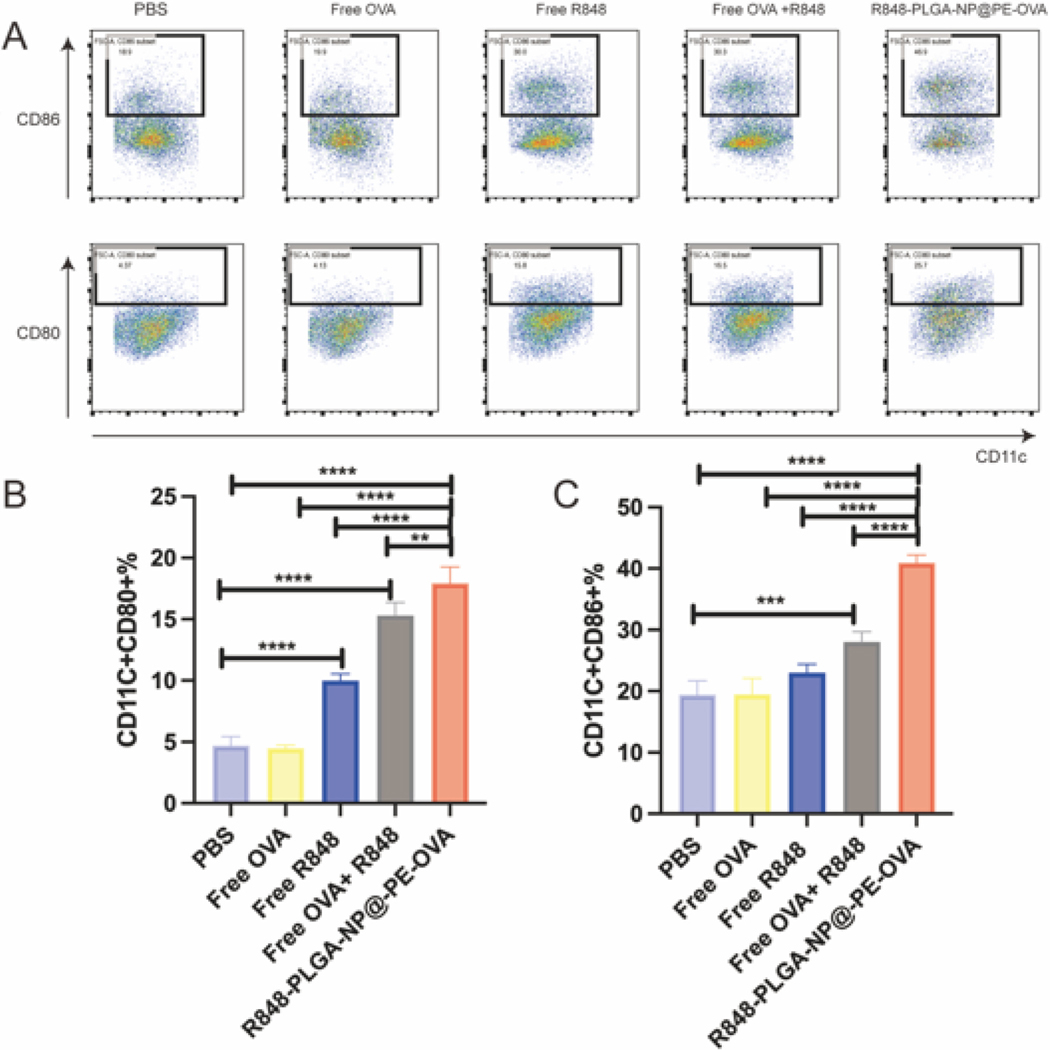
Activation of BMDCs. (A) Representative flow cytometry plots of BMDCs treated with PBS, free OVA, free OVA + R848, and R848-PLGA-NP@PE-OVA for 24 h. (B-C) Quantification of CD80 and CD86 expression following treatment (mean ± SD n = 4). **P < 0.01, ***P < 0.001, ****P < 0.0001.

R848 facilitates the maturation of BMDCs and can enhance antigen presentation. CD80 and CD86 are widely recognized as key surface markers of BMDCs maturation. Accordingly, we assessed the expression levels of CD80 and CD86 on BMDCs to evaluate their maturation status. As shown in Fig. 5, Free R848, Free OVA + R848, and R848-PLGA-NP@PE-OVA treatments resulted in a significant upregulation of co-stimulatory molecules. Notably, R848-PLGA-NP@PE-OVA induced a greater increase in CD80 and CD86 expression compared to Free OVA + R848. Specifically, CD80 expression increased from 15.3 % to 17.9 % (*P <* 0.01), while CD86 expression rose from 27.9 % to 39.9 % (*P <* 0.0001), demonstrating the enhanced immunostimulatory potential of R848-PLGA-NP@PE-OVA compared to free OVA + R848. Interestingly, compared to the Free OVA + R848 group, R848-PLGA-NP@PE-OVA induced a greater increase in CD86 expression relative to CD80, suggesting a stronger enhancement of CD86 upregulation. Moreover, CD86 may play a more critical role than CD80 in the activation of CD4^+^ T cells (Lang et al., 2002), further highlighting its importance in the immunostimulatory effects of the formulation. The superior performance of R848-PLGA-NP@PE-OVA can be attributed to the ability of PLGA nanoparticles to facilitate the sustained release of R848, (Kim et al., 2018). Additionally, the enhanced flexibility and deformability of the Pickering emulsion, compared to conventional emulsions, may contribute to antigen activation by improving its interaction with DCs, thereby facilitating greater antigen uptake and subsequent immune activation (Xia et al., 2018).

### 3.6. R848-PLGA-NP@PE-OVA enhances in vivo activation of DCs

**Fig. 6.**
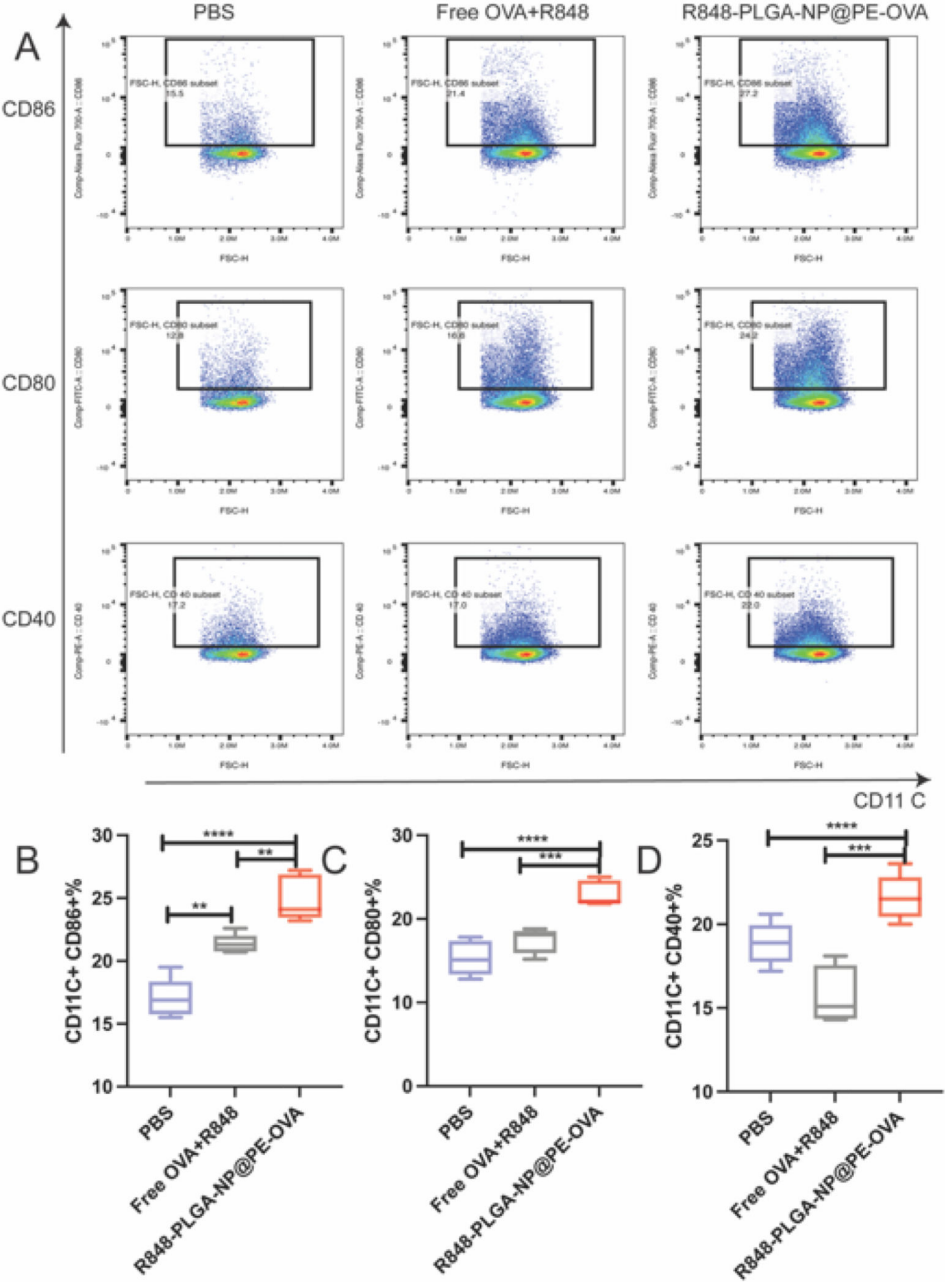
In vivo activation of DCs in spleen following oral vaccination. (A) Representative flow cytometry dot plots showing CD80, CD86, and CD40 expression in splenic DCs after treatment with Free OVA + R848, R848-PLGA-NP@PE-OVA, and PBS. (B-D) Quantitative analysis of DC populations and their frequencies in the spleen (mean ± SEM, n = 5). **P < 0.01, ***P < 0.001, ***P < 0.0001.

To further examine the in vivo immunostimulatory efficacy of R848-PLGA-NP@PE-OVA, splenic DCs were analyzed following in vivo immunization. Gating strategies for in vivo DC characterization are described in Fig. 6A. As shown in Fig. 6B–D, R848-PLGA-NP@PE-OVA–treated BMDCs showed CD40, CD80, and CD86 expression levels that were 1.14, 1.5, and 1.46 times those of the PBS group, respectively (*P <* 0.0001). Moreover, compared to the Free OVA + R848 group the expression levels of these markers were 1.36, 1.32, and 1.16 times those of the corresponding controls, respectivelly (*P <* 0.001). These results are consistent with the in vitro assay, which R848-PLGA-NP@PE-OVA treatment stimulates DC activation.

### 3.7. R848-PLGA-NP@PE-OVA stimulates splenic T cell activation

**Fig. 7.**
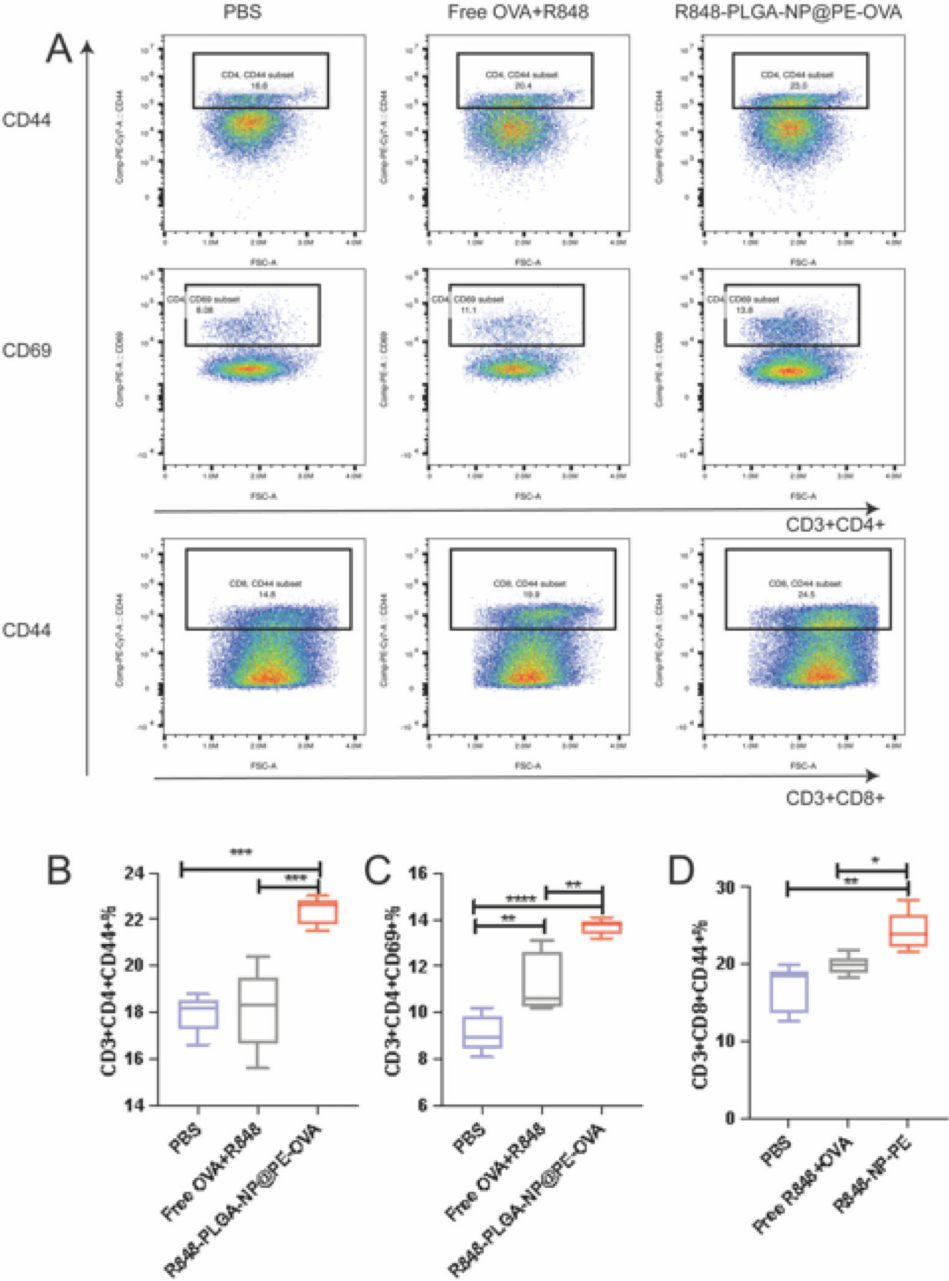
In vivo T cell activation in spleen following oral vaccination (A) Representative flow cytometry dot plots showing CD44, CD69 expression in CD8^+^ and CD4^+^ T cells after treatment with Free OVA +R848, R848-PLGA-NP@PE-OVA, and PBS. (B-D) Quantitative analysis of CD44^+^ CD4^+^ T cells, CD69^+^ CD4^+^ T cells, and CD44^+^ CD8^+^ T cells in the spleen (mean ± SEM, n =5). *P <0.05, **P <0.01, ***P <0.001, ****P <0.0001.

CD4^+^ T cells, which are critical for providing helper functions to enhance CD8^+^ T cell activation and differentiation were investigated. Specifically, we analyzed the CD44 and CD69 expression on CD4^+^T cells to evaluate their prior antigen exposure and early activation status. Gating strategies for T cell characterization are described in Fig. 7A. As shown in Fig. 7B, immunization with Free OVA + R848 resulted in a modest increase in CD44 expression on CD4^+^ T cells compared to the PBS group. In contrast, mice immunized with R848-PLGA-NP@PE-OVA exhibited a significant upregulation of CD44 expression (22.34 %) compared to both the PBS group (17.94 %) and the Free OVA + R848 group (18.1 %) (*P <* 0.0001). Furthermore, as shown in Fig. 7C, immunization with R848-PLGA-NP@PE-OVA significantly increased CD69 expression on CD4^+^ T cells (13.26 %) compared to the Free OVA + R848 group (9.16 %) and the PBS group (11.26 %).

Given the critical role of CD8^+^ T cells in mediating cytotoxic response against infected or malignant cells (Dagotto et al., 2024), we assessed CD8^+^ T cell activation following immunization. CD44 expression on CD8^+^ T cell indicates their potential to develop into memory cells capable of rapid response upon secondary vaccination (Akondy et al., 2017). Therefore, to assess CD8^+^ T cell activation, we measured CD44 expression on CD8^+^ T cells. As shown in Fig. 7D, CD44^+^ CD8^+^ T cell frequency was significantly higher in the Free OVA + R848 and R848-PLGA-NP@PE-OVA groups compared to the PBS group. Notably, the R848-PLGA-NP@PE-OVA group exhibited levels that were 1.4 times those of the PBS group (*P <* 0.01) and 1.2 times those of the Free OVA + R848 group(*P <* 0.1). Combined data suggest that the R848-PLGA-NP@PE-OVA formulation effectively activates both CD4^+^ and CD8^+^ T cells, promoting a coordinated immune response that may improve vaccine efficacy.

### 3.8. R848-PLGA-NP@PE-OVA induces potent NK cell activation

**Fig. 8.**
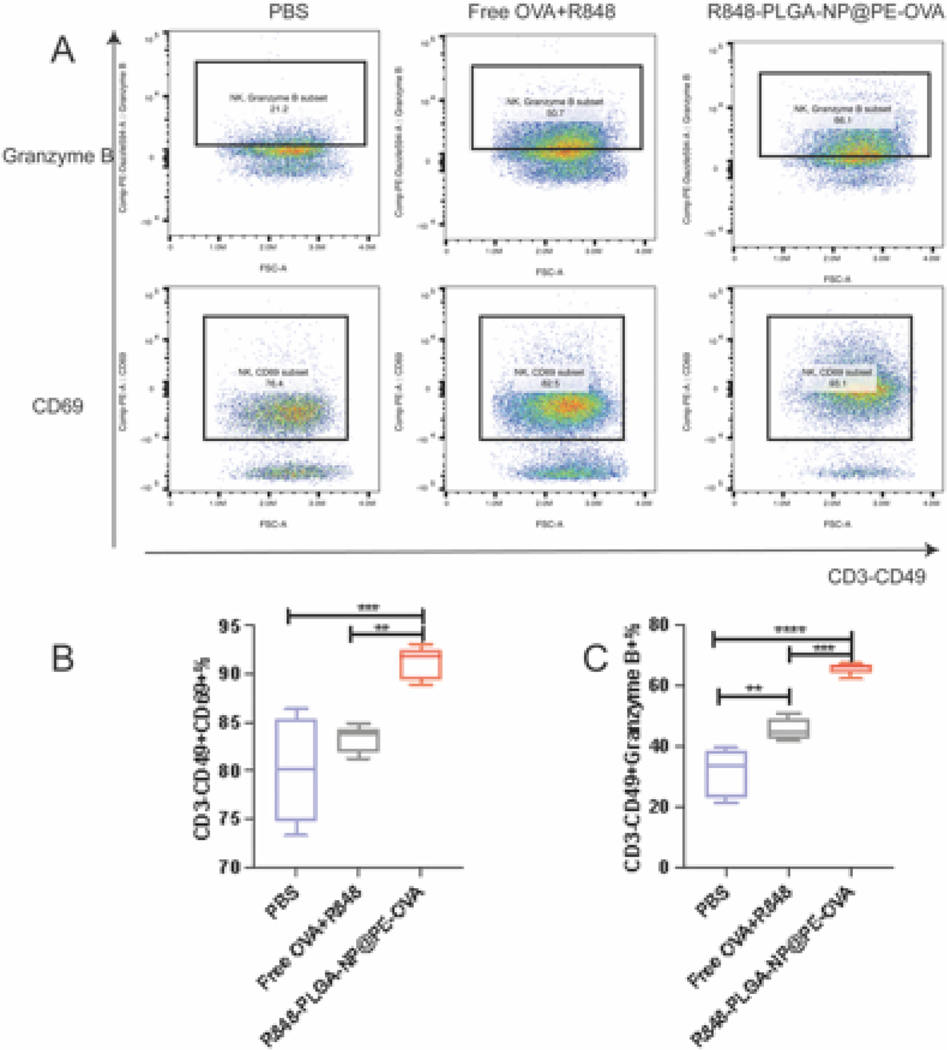
In vivo NK cell activation in spleen following oral vaccination (A) Representative flow cytometry dot plots showing NK cells after treatment with Free OVA + R848, R848-PLGA-NP@PE-OVA, and PBS. (B-C) Quantitative analysis of CD69^+^ and Granzyme B^+^ NK cells in the spleen (mean ± SEM, n = 5). **P < 0.01, ***P < 0.001, ***P < 0.0001.

NK cells, which are innate immune cells with potent cytotoxic function was further analyzed. The gating strategy for NK cell characterization is described in Fig. 8 A. As shown in Fig. 8B, R848-PLGA-NP@PE-OVA significantly increased CD69 expression on splenic NK cells compared to the Free OVA + R848 group (*P <* 0.01) and the PBS group (*P <* 0.001). To further evaluate the functional activation of NK cells, we measured the expression of granzyme B, a critical cytotoxic effector molecule (Fehniger et al., 2007). Consistent with the CD69 results, R848-PLGA-NP@PE-OVA significantly enhanced granzyme B expression (Fig. 8C), reaching 2.1 times the level of the PBS group (*P <* 0.001) and 1.45 times that of the Free OVA + R848 group (*P <* 0.001).

### 3.9. Antigen-specific IgG and IgA responses

IgA is the predominant immunoglobulin on mucosal surfaces and serves as a key marker of intestinal mucosal immunity. In contrast, serum IgG reflects systemic humoral response (Xu et al., 2018). IgA serves as the first line of defense by preventing pathogen invasion at mucosal surfaces (Sheikh-Mohamed et al., 2022), while IgG provides a secondary defense after pathogens breach the mucosal barrier, with levels rising rapidly following secondary immunization (Cerutti, 2010). To assess both mucosal and systemic immunity, we measured IgA levels in fecal samples and IgG levels in serum, that are specific to OVA, the model antigen encapsulated in the oral vaccine.

**Fig. 9.**
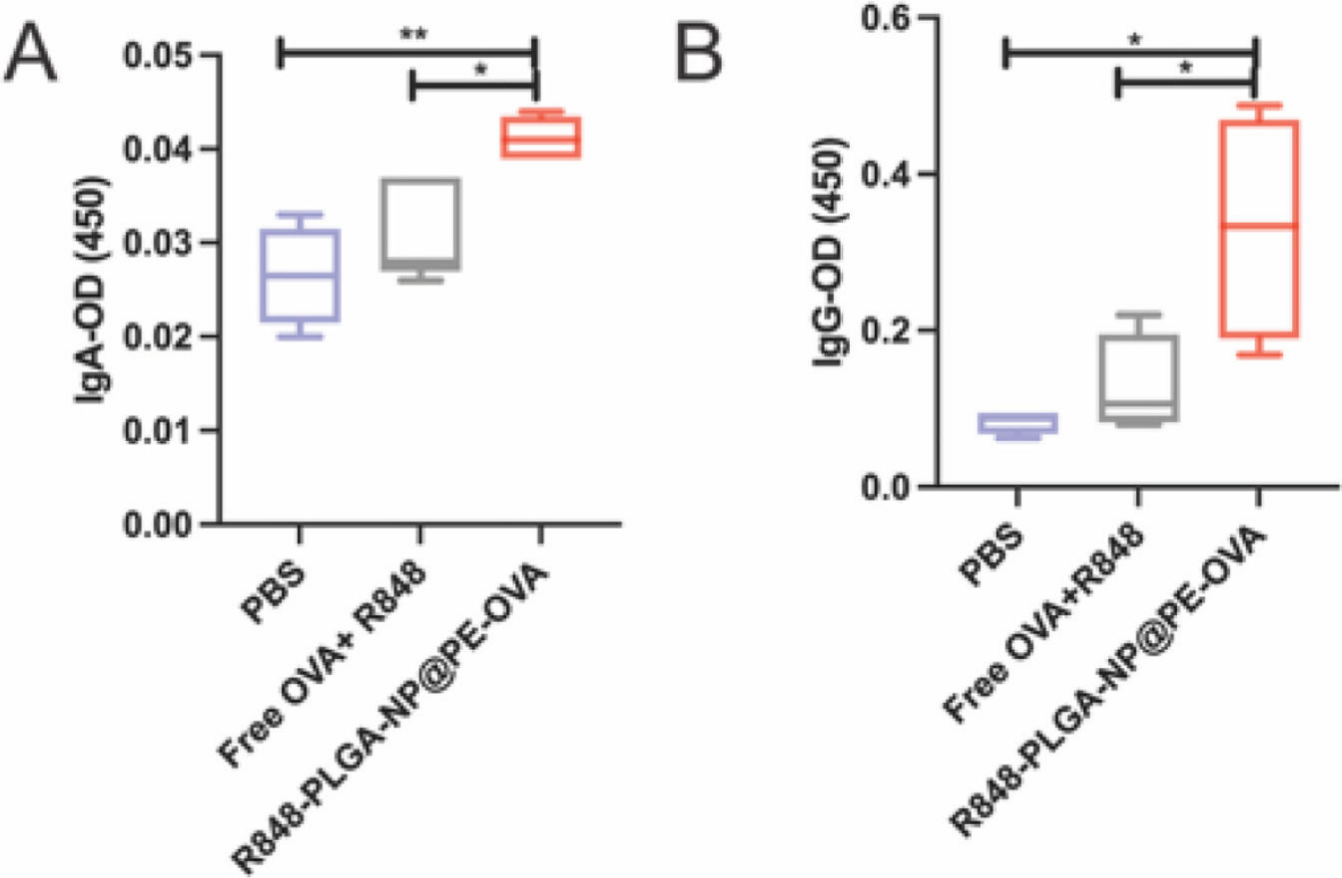
Humoral responses following oral immunization (A) OVA-specific IgA levels in fecal samples. (B) OVA-specific IgG levels in serum. (mean ± SEM, n = 5). *P < 0.05, **P < 0.01.

As shown in Fig. 9A, fecal OVA-specific IgA levels in the R848-PLGA-NP@PE-OVA group were 1.5 times those of the PBS group (*P <* 0.01) and 1.3 times those of the Free R848 + OVA group (*P <* 0.05). Notably, the OVA-specific IgG level in the R848-PLGA-NP@PE-OVA group was approximately 3.9 times that of the PBS group and 2.5 times that of the free R848 + OVA group (*P <* 0.05) (Fig. 9B). The enhanced immune response in the R848-PLGA-NP@PE-OVA group may be attributed to the unique properties of the Pickering emulsion, which offers an enlarged interfacial area that promotes antigen interaction with mucosa-associated immune cells (Du et al., 2022). Moreover, previous studies have shown that small particles achieve more efficient uptake by M cells and enterocytes compared to larger particles, thereby enhancing antigen delivery and immune activation (Gutierro et al., 2002). These findings align with previous research demonstrating that oral vaccines can effectively stimulate both systemic and mucosal immunity by inducing IgG and IgA responses (He et al., 2024) (Carlsen et al., 2023). Collectively, these results demonstrate the potential of the R848-PLGA-NP@PE-OVA formulation to effectively enhance antigen-specific mucosal and systemic humoral immune responses.

## 4. Discussion

An ideal oral vaccine should effectively activate both humoral and cellular immunity to provide comprehensive protection(McNeela and Mills, 2001). Cellular immunity is primarily mediated by CD4^+^ T cells, CD8^+^ T cells, and NK cells, which play essential roles in immune surveillance and host defense. A key mechanism for inducing cellular immunity is the activation of DCs, which facilitate antigen processing and intercellular transfer, enhancing cross-presentation to promote T cell priming and activation (Joffre et al., 2012). Additionally, DCs regulate NK cell activation through direct cell–cell interactions and cytokine secretion, further amplifying the immune response (Altfeld et al., 2011). In addition to cellular immunity, DCs activate B cells and promote antibody production by presenting antigens to follicular helper T cells, driving affinity maturation and class-switch recombination (Krishnaswamy et al., 2018). Hence, DC activation is critical to elicit cellular and humoral immune responses.

In our study, we first evaluated the stimulatory effect of Pickering emulsion-loaded OVA (PE-OVA) on BMDCs. As shown in Supplementary Fig. 5A–B, PE-OVA at an OVA concentration of 100 nM slightly increased CD80 expression; however, the difference was not statistically significant. No notable regulatory effect was observed on CD86 expression. We then conducted in vivo experiments to assess the immunomodulatory effects of PE-OVA on T cells and NK cells. Compared to free OVA, PE-OVA treatment resulted in a slight increase in CD69 expression on CD4^+^ T cells, CD8^+^ T cells, and NK cells (Supplementary Fig. 5C–E), although these changes were not statistically significant. These results suggest that while PE-OVA exhibited a modest immunomodulatory effect, its impact was not substantial, potentially due to insufficient activation of DCs. Therefore, to enhance immune stimulation, we utilized R848 nanoparticles (NPs) as an adjuvant in our study. R848, a TLR7/8 agonist, which triggers activation of downstream signaling pathways that promote DC maturation. Additionally, encapsulating R848 in PLGA nanoparticles provided a particle size favorable for efficient DC uptake, while simultaneously serving as a stabilizer within the Pickering emulsion system for OVA delivery. In this study, Pickering emulsion demonstrated enhanced DC activation in both in vitro and in vivo assays, which co-stimulatory molecule levels were significantly increased compared to PBS and Free OVA + R848 groups. Additionally, antigen-specific fecal IgA and serum IgG levels indicate that DCs were able to efficiently uptake and present antigens to elicit antigen-specific immune responses. Enhanced DC activation may be attributed to several factors including sustained TLR7/8 stimulation by R848 and enhanced antigen uptake, which was achieved by co-delivery of antigen and adjuvant using the Pickering emulsion. Furthermore, the Pickering emulsion system, due to its structural flexibility and adaptability, may increase the contact surface area with DCs, thereby improving cellular uptake and immune stimulation (Du et al., 2024; Yan et al., 2024). By systematically adjusting the oil-to-water ratio and PLGA nanoparticle concentration, we obtained a formulation with improved physical stability, uniform particle size distribution, and high antigen loading capacity. Specifically, a 12:1 oil-to-water ratio combined with 0.4 % PLGA-NP resulted in emulsions that remained stable after storage (Fig. 2). These characteristics are favorable for mucosal delivery and enhance the potential for efficient antigen uptake and immune activation. Our results also demonstrate that the Pickering emulsion remains stable in SGF, suggesting that it may provide protection for OVA and R848, ensuring their stability and bioavailability in GI-tract. This stability is closely associated with the optimized formulation of the R848-PLGA-NP@PE-OVA (Fig. 3 A–D). Consistent with the observed physical stability, the in vitro release studies demonstrated that both R848 and OVA exhibited a slower release from R848-PLGA-NP@PE-OVA compared to their respective free forms in both SGF and SIF (Fig. 3 E–H). These results suggest that the Pickering emulsion not only protected the encapsulated R848 and OVA from premature release in the acidic gastric environment but also achieved sustained release in the intestinal environment, which may contribute to prolonged antigen exposure. Consistently, previous studies utilizing Pickering emulsions for vaccine delivery have reported similar findings (Guo et al., 2025a; Guo et al., 2025b; Peng et al., 2020), indicating that their deformability enhances DC stimulation and immune activation.

Following DC maturation, the next critical step in the immune response is antigen presentation to T cells. CD4^+^ T cells primarily orchestrate immune responses by secreting cytokines and promoting B cell differentiation, ultimately leading to the production of high-affinity antibodies that enhance humoral immunity (Baumgarth, 2021; Cho et al., 2019; Eisenbarth et al., 2021). In our study, immunization with R848-PLGA-NP@PE-OVA significantly increased the expression of CD44 and CD69 in the splenic T cells compared to the Free OVA + R848 group (Fig. 7A–C). CD69, an early activation marker, is known to regulate chemokine expression and facilitate the accumulation of CD4^+^ T cells (Radulovic et al., 2013). Additionally, it plays a crucial role in the generation and maintenance of memory Th lymphocytes, ensuring a more rapid and efficient immune response upon secondary immunization (Schoenberger, 2012; Shinoda et al., 2012). These findings suggest that CD69 contributes to both early CD4^+^T cell activation and long-term memory formation, thereby enhancing immune recall. Moreover, CD44^+^ CD4^+^ T cells support memory Th1 cell activation by promoting effector cell survival (Baaten et al., 2010). CD8^+^ T cells, on the other hand, mediate immune responses by targeting and eliminating infected cells (Collins et al., 2020). In our study, the proportion of CD44^+^ CD8^+^ T cells in the R848-PLGA-NP@PE-OVA group was 1.43-times that of the PBS group (Fig. 7D). The elevated CD44 expression in CD8^+^ T cells indicates their activation and potential cytotoxic function, enabling them to release effector molecules such as perforin and granzyme to eliminate infected cells (Firan et al., 2006; Klement et al., 2018; Seth et al., 1991). In addition to T cells, we observed increased expression of granzyme B and CD69 on NK cells (Fig. 8). Combined data suggests that oral vaccination using R848-PLGA-NP@PE-OVA can elicit a strong cellular immunity.

Humoral immunity, alongside cellular immunity, plays a vital role in vaccine-induced immune responses. Particularly, we measured antigen-specific IgA and IgG responses, that are critical to neutralizing the target pathogens following immunization. Our results demonstrated that OVA-Specific IgG and IgA levels were significantly elevated following R848-PLGA-NP@PE-OVA administration compared to the PBS group and Free OVA + R848 groups (Fig. 9). This indicates that the formulation effectively stimulated B cell activation, leading to a robust antigen-specific humoral immune response. Interestingly, compared to liposome-based oral OVA vaccines reported in previous studies, our formulation induced a more rapid IgG response. In those studies, anti-OVA IgG levels in the plasma were not significantly elevated at day 14 relative to the PBS group, and only showed marked increases at later time points such as days 35 and 42 (Minato et al., 2003). In contrast, our system triggered a statistically significant IgG response as early as day 11. This earlier immune activation may be attributed to enhanced antigen uptake, sustained adjuvant stimulation from R848, and the unique interfacial properties of the Pickering emulsion that promote interaction with mucosal immune cells. Moreover, when compared to delivery systems that also employ Toll-like receptor agonists as adjuvants, our formulation elicited relatively stronger immune responses. For instance, in a previous study, administration of OVA combined with R848 in a nanoemulsion resulted in OVA-specific IgG titers that were less than twofold those of the OVA-alone group (Deng et al., 2025). In contrast, the R848-PLGA-NP@PE-OVA group in our study induced IgG levels that were 3.9-fold those of the PBS group and 2.5-fold those of the Free R848 + OVA group. While differences in experimental design, administration route, and dosing regimens should be considered, these findings suggest that the Pickering emulsion platform may provide a favorable environment for enhancing both mucosal and systemic humoral immune responses.

The enhanced immune response may be attributed to the ability of R848-PLGA-NP@PE-OVA to promote DC activation and antigen-presentation. Compared to the free drug, the solid particle barrier of the Pickering emulsion may protect the vaccine from gastric acid degradation (Li et al., 2023; Sarkar et al., 2019), improving its bioavailability in the small intestine and facilitating mucosal IgA induction (Klein et al., 2014). Moreover, emerging evidence suggests that IgG may collaborate with IgA to maintain intestinal homeostasis (Koch et al., 2016), further amplifying the immune protection. These findings are consistent with previous studies demonstrating that nano drug delivery-based oral vaccine systems enhance IgG and IgA responses by stabilizing antigens, promoting their uptake by APCs, and prolonging immune activation in mucosal tissues (Christensen et al., 2019; Lin et al., 2022; Lu et al., 2023). Therefore, this formulation represents a promising strategy for simultaneously enhancing systemic and mucosal immunity, thereby improving vaccine efficacy.

In addition, oral tolerance remains a major challenge in the development of oral vaccines and is a key focus of our future research. GI tract possesses a natural immune tolerance mechanism designed to prevent excessive immune responses to harmless antigens such as food proteins and commensal microbiota (Tordesillas and Berin, 2018). However, this mechanism may also suppress immune responses to orally delivered vaccines. This form of tolerance is primarily mediated by regulatory T cells (Tregs), tolerogenic dendritic cells (DCs), and various anti-inflammatory cytokines, including IL-10 and TGF-β (Poonam, 2007). To overcome oral tolerance, several strategies have been explored, such as the use of mucosal adjuvants, M cell-targeted delivery systems, and particulate carriers like nanoparticles or emulsions that can enhance antigen uptake and presentation (Faria and Weiner, 2005; Hearn et al., 1996; Liu et al., 2021; Suzuki et al., 2008).

In our study, we developed a Pickering emulsion stabilized by R848-loaded nanoparticles that enhanced dendritic cell activation and antigen uptake. This system may help overcome oral tolerance by providing sustained immunostimulation and co-delivering antigen and adjuvant to promote mucosal immune activation. Thus, it holds promise for improving oral vaccine efficacy. This strategy effectively stimulated both humoral and cellular immune responses, highlighting the potential of this platform to enhance vaccine efficacy. However, for broader clinical application, further investigation is required to assess the long-term safety and biocompatibility, and the implications of its flexibility and deformability on formulation stability. While our study demonstrates its immunogenic potential, comprehensive safety evaluations, including toxicity studies, biodistribution analysis, and long-term immune effects, are necessary to ensure its suitability for human use. Additionally, factors such as large-scale manufacturability, formulation stability under varying environmental conditions, and the impact of different physiological conditions on vaccine absorption warrant further exploration.

Despite these challenges, this delivery system offers a promising approach to improving the stability and immunogenicity of oral vaccines, particularly in resource-limited settings where cold chain logistics and trained healthcare personnel are scarce. By facilitating more efficient vaccination programs, it could contribute to reducing the incidence of vaccine-preventable diseases, decreasing healthcare costs, and ultimately enhancing the quality of life in populations most vulnerable to infectious diseases.

## 5. Conclusion

Pickering emulsion-based oral vaccine delivery system stabilized by R848-PLGA-NP, can elicit both humoral and cellular immune responses. The R848-PLGA-NP@PE-OVA system not only improves DC maturation and antigen presentation but also significantly enhances systemic and mucosal immunity by upregulating OVA-specific IgG and IgA levels. The observed increase in CD4^+^ and CD8^+^ T cell activation, along with the enhancement of NK cells, suggests the potency of R848-PLGA-NP@PE-OVA system to elicit cellular immunity. These findings highlight the potential of PLGA-based Pickering emulsions as an innovative platform for oral vaccine delivery, particularly for mucosal immunization strategies. Furthermore, this approach can be extended to other GI-related vaccines and cancer immunotherapies.

## Supplementary Material

Supplement figures 1-5

## Data Availability

Data will be made available on request.

## Appendix A. Supplementary data

Supplementary data to this article can be found online at https://doi.org/10.1016/j.ijpharm.2025.125890.
