# Supplementary material for: Immunostimulatory Pickering emulsion for oral vaccine delivery
Source: Int J Pharm. Author manuscript; Available in PMC 2025 Nov 25. (PMC12646106; doi:10.1016/j.ijpharm.2025.125890)
Supplement: Supplement figures 1-5 [file NIHMS2112725-supplement-Supplement_figures_1-5.docx]

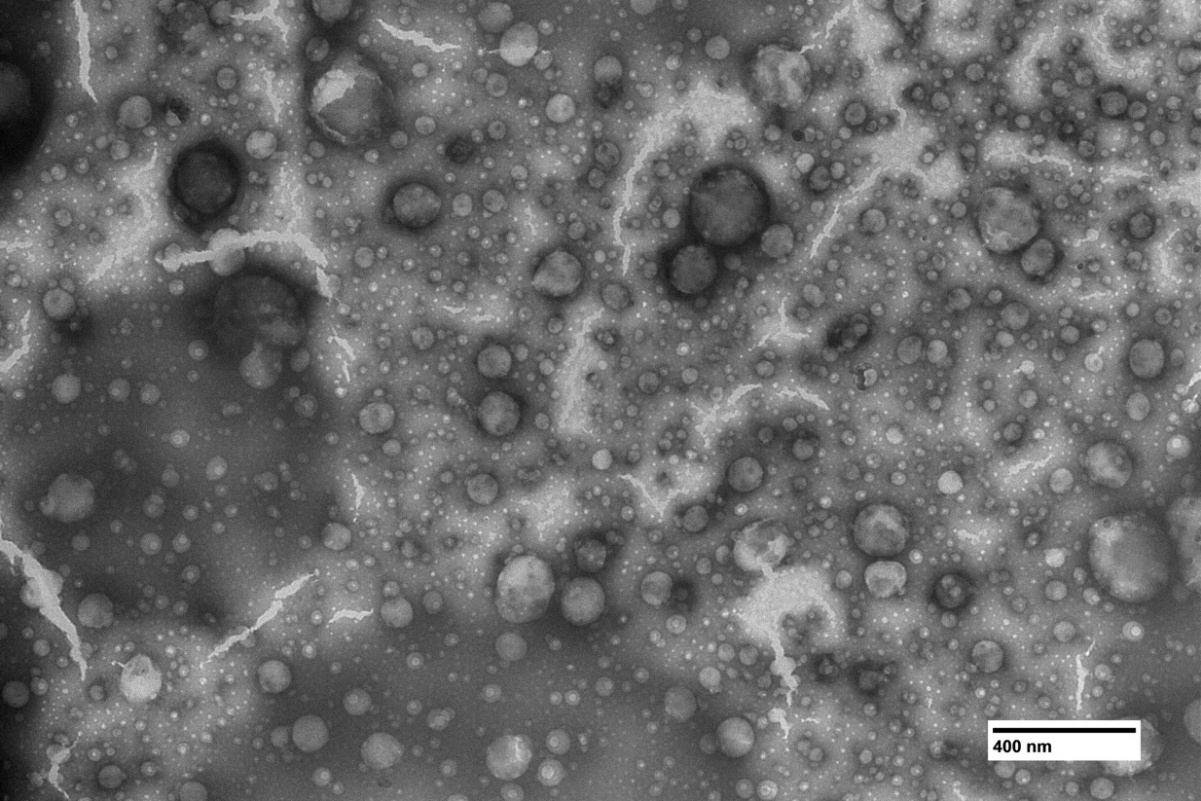


Supplementary Figure 1. TEM morphology of R848-PLGA-NP@PE-OVA


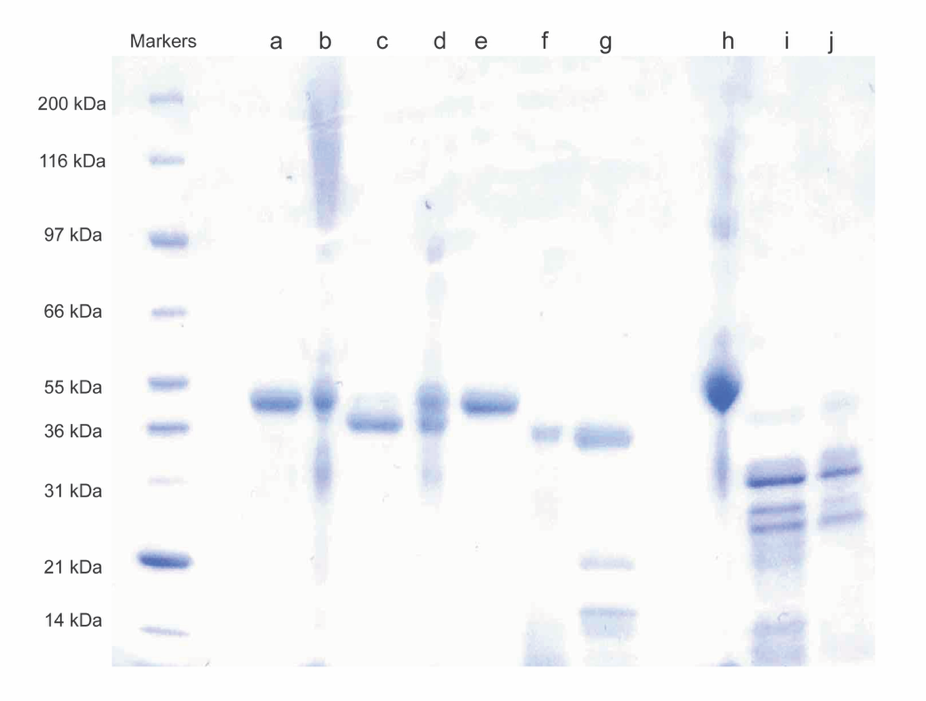


Supplementary Figure 2 SDS-PAGE analysis of free OVA and R848-PLGA-NP@PE-OVA after incubation in SGF (with or without pepsin) and SIF (with or without pancreatin). a: free OV control, b: R848-PLGA-NP@PE-OVA control, c: free ova in SGF without pepsin, d: R848-PLGA-NP@PE-OVA in SGF without pepsin, e: free ova in SIF without pancreatin, f: R848-PLGA-NP@PE-OVA in SIF without pancreatin, g: free ova in SGF with pepsin, h: R848-PLGA-NP@PE-OVA in SGF with pepsin, i: free ova in SIF with pancreatin, j: R848-PLGA-NP@PE-OVA in SIF with pancreatin.

Supplementary Figure 3. The FT-IR spectrum of R848-PLGA-NP@PE-OVA, R848 and OVA.


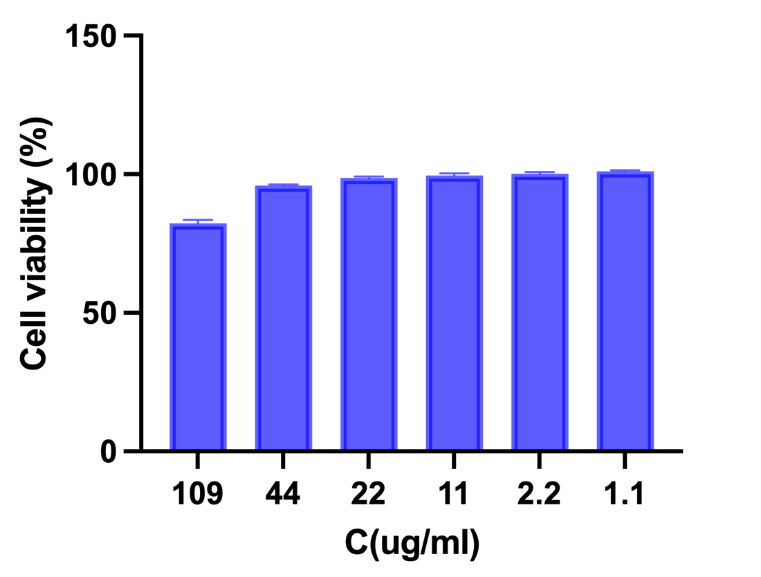


Supplementary Figure 4. BMDC viability following treatment with various concentrations of R848-PLGA-NP@PE-OVA (mean ± SD, n = 4).


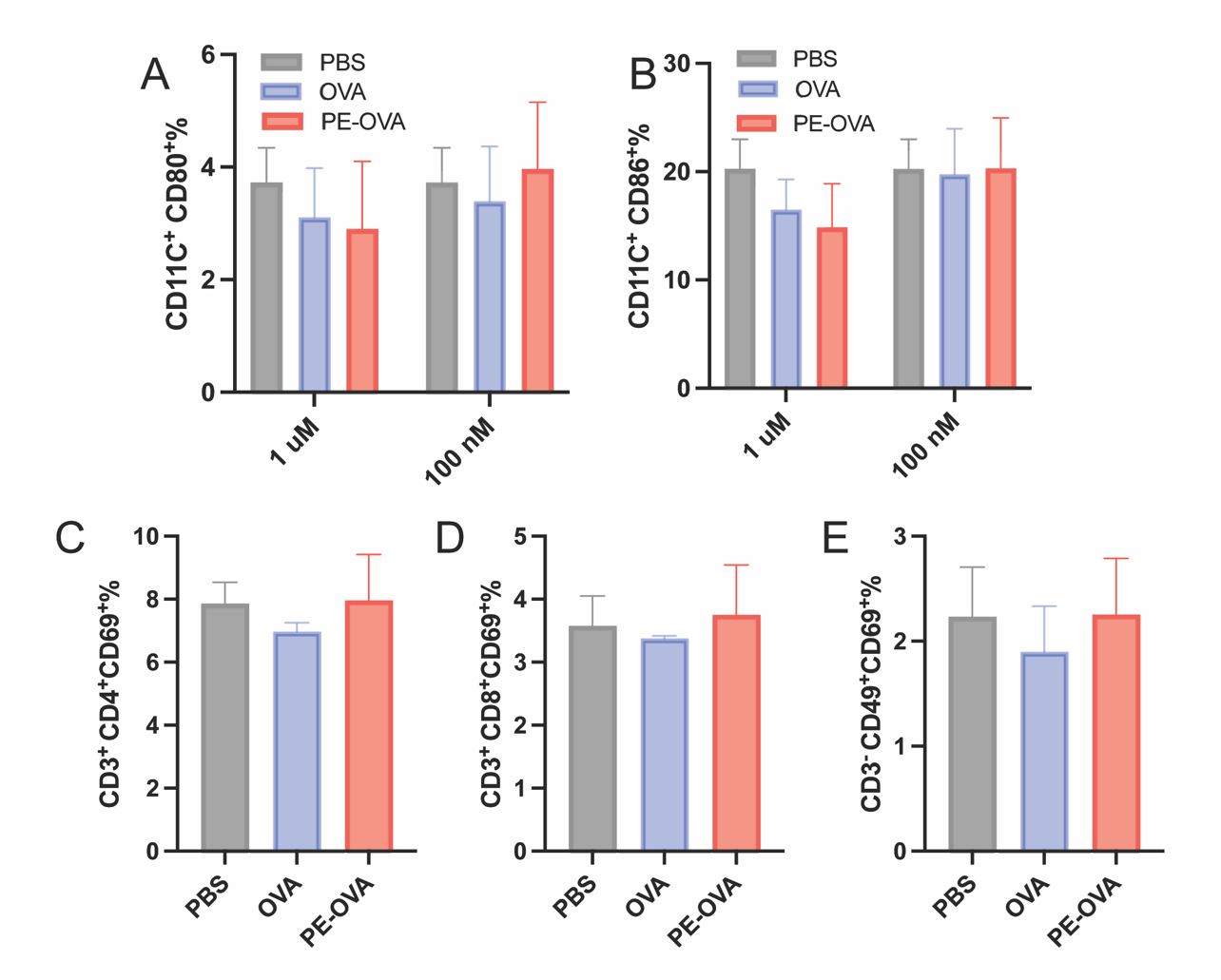


Supplementary Figure 5 (A–B) Activation of BMDCs. Quantification of CD80 and CD86 expression following treatment with PBS, free OVA, or Pickering emulsion-loaded OVA (PE-OVA) for 24 hours (mean ± SD, n = 4). (C–E) In vivo activation of T and NK cells in the spleen following oral vaccination with PBS, free OVA, or PE-OVA. Quantitative analysis of CD69⁺ CD4⁺ T cells, CD69⁺ CD8⁺ T cells, and CD69⁺ NK cells in the spleen (mean ± SEM, n = 5).
